# The immunohistochemical and histologic effects of contrast medium on uterus, fallopian tubes and ovaries, given during hysterosalpingography: rat study

**DOI:** 10.4274/jtgga.galenos.2020.2019.0067

**Published:** 2020-12-04

**Authors:** Eren Pek, Ceren Canbey Göret, Servet Hacıvelioğlu, Gürhan Adam, Mesut Abdülkerim Ünsal

**Affiliations:** 1 Clinic Obstetrics and Gynecology, Dinar State Hospital, Afyonkarahisar, Turkey; 2 Clinic of Surgical Pathology, Sancaktepe Şehit Prof. Dr. İlhan Varank Training and Research Hospital, İstanbul, Turkey; 3 Department Obstetrics and Gynecology, Çanakkale Onsekiz Mart University Health Practice and Research Hospital, Çanakkale, Turkey; 4 Clinic of Radiology, Memorial Şişli Hospital, İstanbul, Turkey

**Keywords:** Free oxygen radicals, hysterosalpingography, iohexol, ionizing radiation

## Abstract

**Objective:**

Previous studies have shown that damage occurs to internal genital tract during hysterosalpingography (HSG). The aim was to show that endometrial and tubal epithelium underwent free radical damage during HSG in an animal model.

**Material and Methods:**

Forty rats were evaluated in five different groups. Two groups received ionizing radiation (15-20 miliRad three times) only. Two further groups received ionizing radiation in combination with iohexol (1-2 mL). The remaining group served as control. Groups were evaluated after seven and forty-two days. Inflammation and cellular changes were evaluated histopathologically. Cellular activity of antioxidant enzymes was assessed immunohistochemically.

**Results:**

Inflammation, and cellular changes were detected at certain rates in all groups (p<0.001). Glutathione reductase, catalase, superoxide dismutase, glutathione S-transferase activities were found to be increased after the HSG (p<0.001).

**Conclusion:**

It is obvious that the cell suffers acute and chronic damage during HSG due to both radioactivity and chemicals. Although there is a lot of research done before, there is no definitive method yet to protect against the harmful effects of iodinated contrast agents and ionizing radiation. So, new methods need to be explored to protect cells and tissues from reactive oxygen radical damage caused by HSG.

## Introduction

Infertility is a condition that prevents the conception of children. The diagnosis of infertility is usually given to couples who have been attempting to conceive for at least one year without success (1,2). It is estimated to affect between 8% and 12% of reproductive-age couples worldwide. In female infertility, approximately 30% to 40% of cases involve ovulatory dysfunction, and 30% to 40% involve tubal and pelvic pathology; 30% of cases are attributed to other unexplained causes, of which reproductive age may be an important contributor (3). Therefore, evaluation of tubal patency and the uterine cavity is important for treatment. Hysterosalpingography (HSG) is one of the oldest imaging techniques. It uses standard X-ray procedures with ionizing radiation and has been used for tubal patency testing for a long time.

It should be noted that this technique has limited accuracy with a positive predictive value 38%. Thus it has been replaced by specific ultrasound procedures, especially with air/saline, foam, and Doppler (4). However, in many places, the original procedure is still a basic method and recommended as the first-line diagnostic tool because it does not require highly qualified practitioners (5,6,7). However, there is clear evidence that the rapidly dividing cells of the reproductive system will be damaged during HSG (8,9). There has been a great deal of research into minimizing the cellular damage to reproductive cells caused by HSG, as well as studies into reducing the negative effects of ionizing radiation and iodinated contrast media on other organ systems (10,11,12,13). However, there is no totally effective method of protection currently available so that minimum doses of ionizing radiation and the use of contrast, only when absolutely necessary, are the mainstays of reducing the deleterious effects of imaging studies. Previous studies investigating the early and late cellular effects of iohexol, an iodinated contrast agent, and ionizing radiation on the uterus, tubes and ovaries have been inconclusive. Given that HSG is a diagnostic method used in women who already have low reproductive capacity further clarification of these effects is important. The aim of this study was to investigate the effects of HSG, with and without iodinated contrast, on endometrium, tubes and ovarian epithelial cells in an animal model.

## Material and Methods

This study was performed in the Laboratory Center for Experimental Studies of Çanakkale Onsekiz Mart University with the approval of the University’s Laboratory Animals Ethics Committee (approval number: 2016/01-02).

**Animals:** Forty female Wistar albino rats, aged 12-14 weeks, with regular cycles and weighing 250-300 g were kept under a 12-hour artificial light/dark cycle at a temperature of 20-24 °C. The animals were kept in groups of five per cage, and were fed with standard pellets and tap water. All rats in the estrous cycle (estrus phases of rats were confirmed by vaginal cytology) were randomly divided into five (n=8) experimental groups designated A, B, C, D and E. The number of rats in each group was set as the minimum number that could be statistically significant to prevent animal wastage.

**Chemicals:** Clinical substances were obtained from GeneTex glutathione S-transferase pi 1 antibody GSTP1 GTX31766-100 (GeneTex, Inc. CA/USA), GeneTex glutathione reductase antibody N2C2 GTX114199-100 (GeneTex, Inc. CA/USA), Novusbio superoxide dismutase antibody SOD1 NBP224915 (Novus Biologicals, LLC. CO/USA) and Novusbio catalase antibody CAT NBP2-24916 (Novus Biologicals, LLC. CO/USA). DAKO (Agilent: Chemical Analysis, Life Sciences, and Diagnostics, Santa Clara, CA/USA) automatic dyeing machine was used for immunohistochemical staining. Iohexol (Omnipaque 350 mg/100 mL, Opakim Medical Products Industry and Trade Corporation. İstanbul /Turkey) was used as radiocontrast medium.

**Experimental design:** The procedural steps were performed as detailed in Table 1.

**Figure 1:**
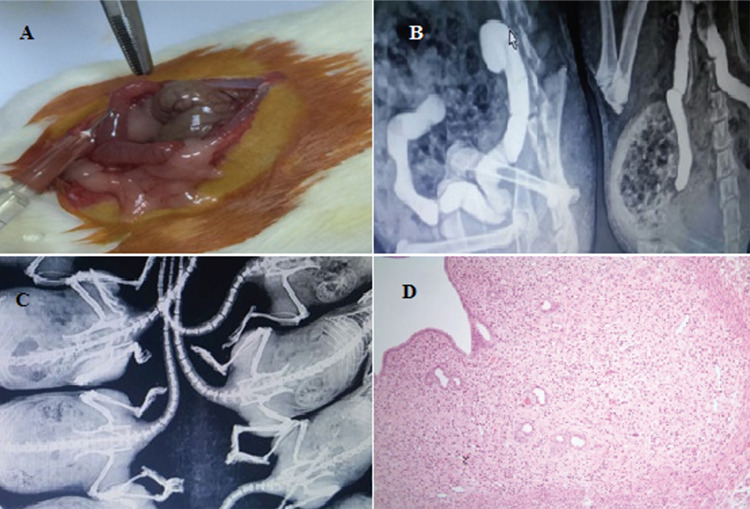
(A) Uterine injection of radiocontrast material from the rat’s cervix with a tuberculin injector. (B) The hysterosalpingography image after the iohexol injection. (C) The hysterosalpingography image which the group without radiocontrast agent. (D) The normal appearance of endometrium and columnar epithelium and glandular epithelium (hematoxylin-eosin staining, x40) (the image of histological section of group A)

**Surgical, radiation and iohexol application procedure:** 400 mg/kg/intraperitoneal dose of chloral hydrate was administered for anesthesia (8). On the first day, after skin cleaning the rats with 10% batticon, a midline incision (approximately 2 cm) was made to access the abdominal cavity. This incision process was applicable to all groups. Then eight rats were directly sacrificed, and the uterus, tubes, and ovaries were removed (group A). In groups C and E, subsequent to incision, 1-2 mL of iohexol was introduced via an injector to the animals’ cervix (Figure 1A). Next, the other groups, with the exception of group A, were exposed to radiation (Figure 1B, C). In groups B and D all-body irradiation was applied at a dose of 15-20 miliRad three times with 3-minute intervals to the rats after opening of the abdomen. Groups C and E also received three doses of total body irradiation; the first dose radiation was given while iohexol was injected and then the other two doses were given in the same way as for groups B and D.

The abdomens of the rats in all groups were closed continuously using absorbable suture materials (4.0 vicryl-rapide) after surgical procedures were completed. For the evaluation of acute (early) effects, after seven days laparotomy was performed again in groups B and C. In groups D and E repeat laparotomy was performed after forty-two days for the evaluation of chronic (late) effects and the uterus, tubes and ovaries were removed.

**Preparation for pathological and immunohistochemical evaluation:** The pathological materials were preserved in 10% formaldehyde and fixed. Two different histological preparations were undertaken. Sections with a thickness of 3-5 microns were prepared from all tissues removed. One group of tissue sections were stained with hematoxylin and eosin for histopathological evaluation under the light microscope. The other group of tissues were embedded in paraffin and 4-5 micron-thick sections were taken for immunohistochemical examination. Sections were stained with antibodies specific for glutathione S-transferase, glutathione reductase, superoxide dismutase and catalase.

**Histopathological scoring:** Vascular ectasia, inflammation, and epithelial cytological and architectural features were evaluated and scored using objective criteria by the same pathologist (8,14,15). Epithelial architectural features; (a) tufting, (b) stratification, (c) chromatin disorganization, (d) irregularity in nucleus contour, (e) increases in nucleus size and ratio of nucleus/cytoplasm, (f) pleomorphism, (g) presence of nucleoli, (h) mitosis and (i) hyperchromasia. All the criteria evaluated except inflammation and vascular ectasia were reported as cellular changes. A minimum of five fields were evaluated on each tissue slide with 40 and 400 magnification and assigned scores for severity of changes as follows: no effect or no staining (0), mild effect or poor staining in localized areas (1), the presence of moderate influence or moderate staining (2) and severe effect or strong staining (3). The other sections which were prepared for immunohistopathological assessment (glutathione S-transferase, glutathione reductase, superoxide dismutase, and catalase) were again evaluated by the same pathologist and the same scoring system was used.

### Statistical analysis

For statistical analysis, SPSS version 20.0 was used (IBM Inc., Armonk, NY, USA). Descriptive statistics was used to calculate the mean scores and standard deviations of each evaluated histopathological finding. (mean ± standard deviation). Statistically, the Kruskal-Wallis H-test was used to determine whether mean differences were significant in terms of group variables, and Mann-Whitney U test was used to determine the group from which the differences originated. Significance was accepted at p<0.05.

### Iohexol, ionizing radiation and measured antioxidant enzymes

Understanding the mechanism of damage caused by ionizing radiation and iodinated contrast media to cells and tissues will be useful here. Iohexsol is a water-soluble, non-ionic, monomeric and low-osmolarity iodinated contrast media. It may show direct cellular toxicity or it may cause indirect toxic effects through the formation of reactive oxygen radicals. This effect is mediated by the release of vasoconstrictor substances such as adenosine, endothelin, vasopressin, angiotensin 2 and dopamine. The result is hypoxia and the release of free oxygen radicals (16,17). Osmolarity is thought to be an especially important factor in these effects. However, osmolarity alone is insufficient to explain this situation. The osmolarity of mannitol is similar to some iodinated contrast agents, but the pathological effects are not the same (18). Thus it is useful to ask whether the use of antioxidants and vasodilating agents are protective against this effect? Many studies have investigated this question. Melatonin, L-carnitine, vitamin C, vitamin E, amifostine, amlodipine, curcumin, N-acetylcysteine and trichloroacetic acid have all been studied (8,9,10,11,15,19,20,21,22,23,24). The effects of radiation on living tissues can be divided into four stages. In the first step, energy is transferred to the substance. This stage is known as the physical step and causes ionization of the substance. The products that appear after the first stage are unstable and cause reactive products. This second stage is the physico-chemical stage. In the chemical stage, the third stage, reactive products interact with cellular structures. The result is production of free radicals. The biological step is the final stage and starts with enzyme reactions that cause a variety of damage, including DNA molecular damage. However, some of this damage can be repaired. Damage that cannot be repaired leads to cell death (25). Physico-chemical changes caused by ionizing radiation in the cell are very rapid (less than a second). In contrast, it may take hours, days, months or even years for biological results to occur. Ionizing radiation causes the breaking of chemical bonds in intracellular molecules, especially chromosomes. If this genetic damage, including de novo mutations, are not corrected by repair mechanisms, they can lead to apoptosis. However, if there is no cell death, they may result in cancer at some point in the future. The effects from energy absorption are direct effects. On the other hand, there are indirect effects that occur through the formation of free oxygen radicals (26,27). Water molecules maybe ionized when the cell is exposed to radiation. A positively charged water molecule and free electrons are formed. Free electrons combine with another water molecule to form a negatively charged water molecule. Positive and negative water molecules are unstable and break down to form ions and free radicals (28,29,30). Cells with higher reporoductive turnover, such as those found in the genital system and reproductive cells, are more sensitive to radiation. Cells are most susceptible to cell death during the G2 stage and mitosis (31,32). The first response to oxidative stress from the cell is through antioxidant enzymes. The most important enzymes in this response are glutathione peroxidase, superoxide dismutase, catalase and glutathione reductase. Other non-enzymatic defenses include antioxidant compounds such as vitamin E, vitamin C, beta carotene, transferrin, ceruloplasmin, haptoglobin and albumin (33,34). The most important enzymic activity is catalyzed by superoxide dismutase which breaks down superoxides. When superoxides are broken down, hydrogen peroxide is formed (35) and catalase will inactivate hydrogen peroxide (36). During these events, glutathione S-transferases act as catalysts (37,38). Glutathione reductase is an enzyme that converts oxidized glutathione, which occurs during reactions catalyzed by glutathione S-transferase, to reduced glutathione (39). Therefore, the aim in this study was to measure the activity of each of these enzymes in the cell using immunohistochemical staining.

## Results

Considerable cellular and histopathological changes were observed for all other groups and criteria when compared to the control group.

First, the normal glandular and columnar epithelium sections of the control group were examined. Figure 1D shows a normal section from the control group. The entire assessment was carried out by the same pathologist. The status of the basal metabolic activity of a normal cell from the control group was evaluated immunohistochemically and histopathologically. This was used as a baseline for comparison and was assigned a score of “0” score, corresponding to the “no effect or no staining” condition.

**Figure 2:**
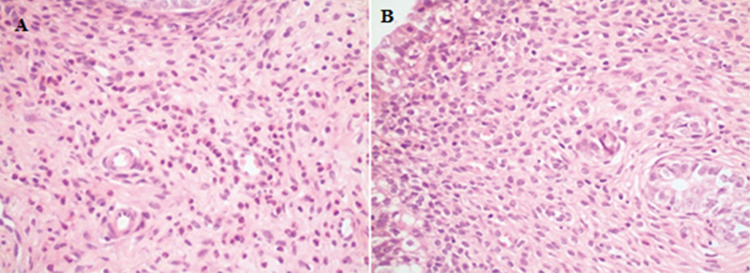
(A) Mix type intense inflammation in endometrial stroma; mainly consisting of the eosinophilic leukocytes (group B) (hematoxylin-eosin staining, x400) (B) mild reactive changes and mild inflammation in superficial cells (group E) (hematoxylin-eosin staining, x400)

**Figure 3:**
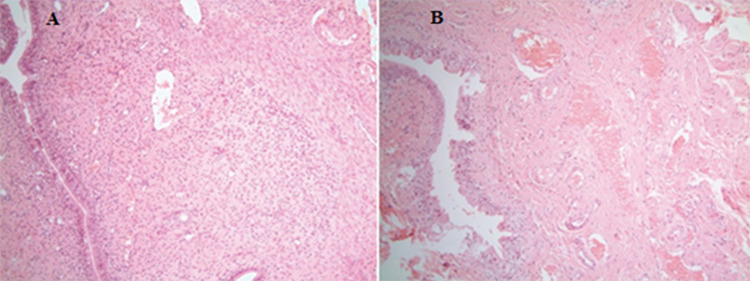
(A) Reactive changes in superficial cells, mix type of mild inflammation in the stromal areas and moderate vascular ectasia (group D). (Hematoxylin-eosin staining, x400) (B) intensive vascular congestion, mix type of mild stromal inflammation, reactive changes in superficial cells (group C). (Hematoxylin-eosin staining, x40)

**Inflammation:** Inflammation was detected intensely in all groups and a statistically significant difference was found between scores for the experimental groups compared to the control group (p<0.001). Ionizing radiation-induced inflammation was more evident in the acute phase (group B) than in chronic phase (group D). As a result of the histopathological evaluation of inflammatory changes using numerical scoring system, it was determined that group C received the highest scores arithmetically. Thus, inflammation scored higher in the early stage of conditions where the cell was exposed to ionizing radiation with together iohexol (Figure 2A, B and 3A, B).

**Cellular changes:** When compared to the control group, major changes were observed in the structure of cells in all groups (p<0.001). The most severe changes were seen in the chronically exposed animals’ tissues. When each group was evaluated separately it was striking that iohexol increased the deleterious effects of ionizing radiation (Figure 2A, B).

**Vascular ectasia:** The results were statistically significant in all other groups compared to the control group (group A) (p=0.009). Vascular ectasia was more evident in group C. (Figure 3A, B).

**Immunohistochemical evaluation results:** The activity of all antioxidant enzymes were increased in animals exposed to both iohexol and ionizing radiation. In addition the effects were more marked in chronic exposure animals compared to acutely exposed animals.

**Figure 4:**
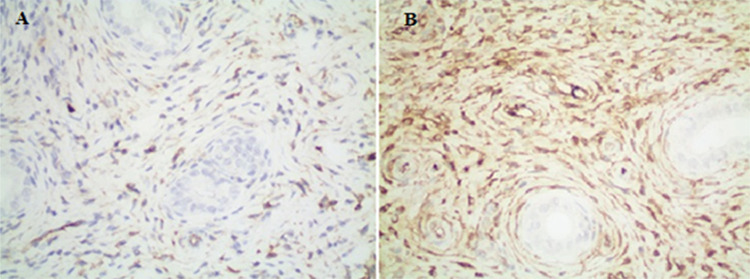
(A) Mild immunohistochemical positivity seen that when the tissue stained with glutathione reductase (group B). (Immunohistochemical glutathione reductase staining, x400) (B) severe immunohistochemical positivity with glutathione reductase (group E) (immunohistochemical glutathione reductase staining, x400)

**-    Glutathione Reductase:** Glutathione reductase activity was observed to be increased at different rates in all groups compared to the control group as a result of immunohistochemical examination (p<0.001) (Figure 4A, B).

**Figure 5:**
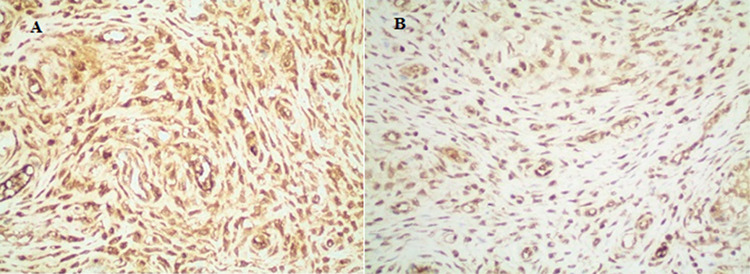
(A) Immunohistochemically severe positivity with Catalase (group E) (immunohistochemical Catalase staining, x400) (B) immunohistochemically severe positivity with superoxide dismutase (group D). (Immunohistochemical superoxide dismutase straining, x400)

**-    Catalase:** For catalase activity, we found significant histological differences in all groups to compared with the control group (p<0.001) (Figure 5A).

**-    Superoxide dismutase:** Superoxide dismutase activity was more intense in all study groups rather than control group (p<0.001) (Figure 5B).

**Figure 6:**
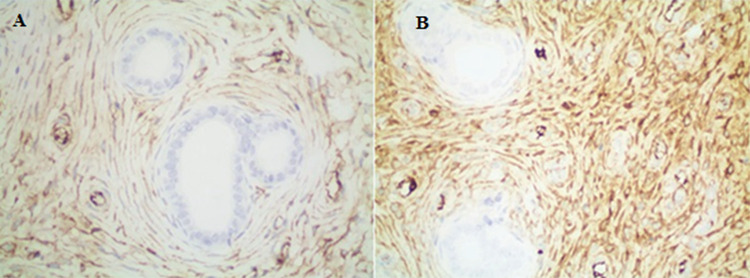
(A) Immunohistochemically moderate positivity with Glutathione S-transferase (group B) (immunohistochemical Glutathione S-transferase staining, x400) (B) immunohistochemically severe positivity with Glutathione S-transferase (group E) (immunohistochemical Glutathione S-transferase staining, x400)

**-    Glutathione S-transferase:** When all groups were compared with the control group, we detected increased changes at different intensities (p<0.001) (Figure 6A, B).

**Interpretation of results (Table 2):** Group E exhibited the lowest levels of inflammation compared to the other experimental groups, apart from control animals. This should be no surprise as the first response of the cell to trauma is inflammation among the parameters which we evaluated. At the end of the inflammatory process, the cell will either rescue itself, or undergo apoptosis or a necrotic uncontrolled process. Groups E and D contained the chronically exposed animals. Therefore, the expected result is a greater degree of inflamation in the acute groups - groups C and B. This finding was confirmed in our study.

Vascular ectasia was more dense in the acute period, in a similar fashion to greater inflammation. This may be due to vascular ectasia and congestion being a part of the inflammatory process.

As can be seen from Table 2, anti-free oxygen radicals enzyme activity is more intense in the late (chronic) period. The presence of iohexol is an additive factor to the formation of free oxygen radicals caused by ionizing radiation. As a result the greatest cellular damage was found in the chronic groups and was more marked in animals exposed to both iohexol and ionizing radiation compared to those only exposed to the radiation.

## Discussion

HSG, which should be done in the follicular phase of the cycle, evaluates the contour of the uterine cavity, cervical canal, and tubal lumina. It is one of the basic tools for infertility diagnosis and has become a standard test for evaluation of infertility worldwide. This procedure is also useful for evaluation of Mullerian system anomalies, recurrent pregnancy losses, abnormal uterine bleeding or amenorrhea and cervical insufficiency. During HSG contrast material is injected into the uterus and this material migrates into the fallopian tubes. Then a series of X-rays, or fluoroscopy is performed. The contrast material shows white in the images allowing any abnormality of structure to be detected. However, the short- and long-term effects of ionizing radiation and contrast medium on tissues are not known. The results of this study show that HSG, a widely used diagnostic technique, may lead to cellular injury and damage to reproductive tissues. Imaging methods using ionizing radiation play an important role in the early diagnosis and treatment of diseases. In diagnosis and treatment, there is the possibility of radiation-induced damage to the patient despite the radiation dose being kept as low as possible and radiation precautions being taken (40). There is no realistic prediction of the diversity and size of health problems that will occur in living organisms at low doses (≤10 cGy) of ionizing radiation. In this respect, low-dose ionizing radiation should not be viewed as safe or tolerable under any circumstances because, due to radiation and independent of the dose, somatic mutations may develop that may lead to neoplastic and non-neoplastic diseases (41). Ionizing radiation may directly and/or indirectly produce various effects in DNA by reactive free radical production (42,43,44). The effects of ionizing radiation can occur in two different ways; stochastic or deterministic effects. Stochastic effects are independent of the exposure dose. It may even occur at very low doses (40). These types of damage are important subsequent to HSG. In addition, the presence of other factors, such as the total radiation dose received, cellular defence mechanisms, the dose given in each session, the duration of exposure, simultaneous chemicals, and other factors that may lead to proto-oncogen activation, increases ionizing radiation-related damage. When cells are exposed to ionizing radiation during mitosis and the G0-G1 phases, the frequency of unstable dicentric chromosomes increases and chromosome aberrations may occur, due to incorrect regulation of chromosomal fragments (40,45,46,47). In this respect, even low-dose ionizing radiation should not be viewed as safe or tolerable under any circumstances. Any exposure to ionizing radiation, independent of dose may eventually result in neoplastic change or non-neoplastic disease. DNA alterations and breaking to double-strand occur in cells exposed to ionizing radiation. Activation of phosphorylase and kinases prevent DNA repair, and consequently the G1, S, and G2 cell cycles cannot proceed, leading to cellular death through various mechnisms including apoptosis, mitotic catastrophy, and terminal binding (48). HSG is done in the proliferative phase of the cycle, in order to sure that the woman is not pregnant when the procedure is performed. So, basal cells which will start mitosis, will be more affected by radiation, during this process. Analysis methods at the cell and tissue level are very important to explain possible early and late effects of radiation on different tissues. The total radiation exposure to the patient during HSG withdrawal was calculated as 713 cGy/cm^2^ (range: 247-1,623 cGy/cm^2^) by Fernández et al. (49) In another study, the average radiation dose exposure of the reproductive organs during an HSG procedure was found to be 500-1000 mRad (50). An avarage human being has 17,000-20,000 cm^2^ surface area and a rat that weighs 250-300 g has 300-400 cm^2^ average surface area. In line with these previous studies, we determined the dose that should be applied to rats as 15-20 mRad (9). The development of the first follicular wave in the rodents to the antral follicle occurs in about three weeks. The developmental stage of primordial follicle to secondary follicular may take >30 days (51). Well-developed secondary follicles are observed on the seventh day (52,53).

Pala et al. (9) showed that HSG treatment caused a significant increase in epithelial degeneration in the rat endometrium at three hours after HSG withdrawal. Lee et al. (54) investigated the primary and primordial follicular damage after exposure to gamma radiation in rats and found that the most significant damage occurred after three hours with a reduction after 6-12 hours following radiation exposure. However, Can et al. (19) chose a period of three hours to examine possible acute radiation damage and a period of one month to investigate chronic effects. Our aim was to go further than previous studies. For this reason, we chose a period of seven day to examine possible ionizing radiation and contrast medium damage in the early “acute” period and a six-week period to investigate possible late period ionizing radiation and contrast medium damage. However, for the first time, an experimental model of the HSG process taking the effects of iohexol and ionized radiation separately, on ovarian, fallopian tube and endometrium histopathology are studied. This is the first pilot study in this area and we believe it is a strength of our study. Although, there have been previous studies investigating the damage caused by iodine contrast medium on cells and tissues, especially the renal tubular system, there is no study that clearly shows its effects on the female internal genital system. Therefore, the effects of iohexol are not as clearly elucidated as those of ionizing radiation. Our current knowledge is limited to research done on other systems. Solomon and Dauerman (55) have investigated the mechanism of action of iodinated contrast agents on the renal tubular system. These authors reported that the mechanisms responsible for the pathogenesis of contrast induced nephropathy are thought to be a combination of the direct tubular toxicity of contrast media, reduction in medullary blood flow, and generation of reactive oxygen species (ROS), in which ROS play a central role (55). ROS can cause vascular endothelial injury and may further intensify tissue parenchymal hypoxia by causing endothelial dysfunction and dysregulation of membrane transport (56,57,58). Iohexol has high viscosity and osmolality among the low-osmolality contrast media and these characteristics both contribute to cell and tissue toxicity. Iohexol decreases extracellular volume contraction. The direct vasoconstrictor effects of iohexol and further exacerbation of ischemia are significant because the vasoconstricting molecules, including renin, endothelin, and adenosine, increase and the vasodilatory molecules such as prostaglandin and nitric oxide decrease (59). Iohexol is a non-ionic, monomeric, iodinated contrast medium (ICM). Heinrich et al. (60) compared different contrast media with the tetrazolium-based colorimetric assay, and when ICM were compared at equal iodine concentrations (75 mg I/mL), they found that dimeric contrast media showed a slightly weaker effect on inhibition of mitochondrial dehydrogenases than monomers, but this difference was not statistically significant. In the same study, the contrast media were also compared at molar basis and it was shown that dimeric ICM were significantly more cytotoxic than monomers on cultured renal cells (60). Carlisle et al. (61) exposed embryonal cancer cells to iohexol, iopamidol and metrizamide at concentrations below those used for clinical myelography and investigated the outcome using light and electron microscopy. They reported cytologic changes, consisting of swelling and vacuolation of mitochondria and other cytoplasmic organelles, which were observed within one hour of exposure to the contrast media. They observed that after 12 hours, there were changes in shape of cells and cell death. They repeated the study in neuron cultures derived from embryonic stem cells and rat dorsal stem ganglion cell cultures. They reported that iohexol and other iodine contrast media are cytotoxic to cells in culture at less than 20% of the concentration used for myelography and this could contribute to the adverse reactions to myelography seen in people and animals (61). Jensen et al. (62) in their studies with water soluble iodinated contrast agents, the iso-osmolal contrast medium iodixanol was found to be less toxic than iohexol in cultured cells of rat proximal tubule origin. It may be thought that these results suggest that using iodixanol during HSG may be more beneficial but it should not be forgotten that iodixanol is a dimeric form of ICM. Berg et al. (63) showed that iohexol, iodixanol, ioxaglate and diatrizoate all possess antioxidant properties in vitro. The reason for this is unknown, but it is possible, although speculative, that the antioxidant properties of the ICM may contribute to the lower cell death at early time points (63). The results of our study likewise show that all effects, except inflammation, are more severe in the late period. More studies will be required to reach a firm conclusion in this matter. Our results were similar to studies in which ionized radiation was evaluated alone, and ionized radiation together with iodinated contrast agents was evaluated in a particular part of the female genital system, or tissues of other systems. As seen from all studies, both ionizing radiation and iodinated contrast agents cause harmful effects at the cellular level. The results we obtained in our study were in agreement with earlier findings. The point we want to emphasize is that all cellular changes, except inflammation and increased vascularization following inflammation, are present to a greater degree and more severe in the late period. Combined ionizing radiation and iohexol produced more severe cellular changes and a greater increase in reactive oxygen radicals in the tissues examined in our study. The question that needs to be considered here is the necessity of finding methods to protect the reproductive cells of infertile-subfertile women, or to ensure minimal damage during HSG. To mitigate the deleterious effects antioxidant substances may be applied during the process, as in many studies where ionizing radiation and iodinated contrast agent are used together. Pala et al. (9) investigated vitamins C and E for the prevention of endometrial cell damage induced by HSG and reported some success. Yılmaz et al. (20) also contributed to the literature with their studies that pre-HSG melatonin use can protect on ovarian surface epithelium. Gülle et al. (22) proposed different approaches with two different studies. They reported that L-carnitine or Curcumin may be beneficial to protect against the negative effects of ionizing radiation on ovaries (21,22). Can et al. (19) used amifostine to prevent ovarian damage caused by ionizing radiation. Yurut-Caloglu et al. (23) compared the protective roles of L-carnitine and amifostine against radiation-induced acute ovarian damage. Sapmaz et al. (15) examined the effect of trichloroacetic acid attachment and instillation methods on dysplastic changes in ovarian surface epithelium. However, it was reported that more research and studies should be done in all these studies. Kilciksiz and Demirel (11) performed a study using N-acetylcysteine to prevent the negative effects of ionizing radiation and oxidative stress. Karaman et al. (10) reported that, as a novel approach, agomelatine can be used to prevent nephrotoxicity caused by the use of iodinated contrast media. Duan et al. (24) investigated the protective effect of amlodipine to nephrotoxicity of high- and low-osmolar contrast media. Also, research has been undertaken in the urinary system with the use of theophylline, sodium bicarbonate or similar materials. These findings may be of some relevance in the genital system as there is a shared embryological origin with the urinary system (64). To our knowledge, no proven benefit has been found for the use of other renal protective agents such as N-acetylcysteine, sodium bicarbonate, diuretics, and theophylline in the genital system (65). Sapmaz and Akpolat (8) used lipiodol (iodinated ethyl esters of fatty acids of poppyseed oil) in their studies as a different approach. Lipiodol significantly reduced dysplastic modifications and increased fusiform structures in the myometrium. Lipiodol plus melatonin restored all the negative changes (8). Lipiodol is a water-insoluble iodinated contrast media. It is possible to use both oil and water soluble contrast media during HSG. There are a few differences between the two contrast agents in the evaluation of intra-uterine pathology and in the evaluation of the tubal patency (66). Lipiodol contains mostly linoleic acid and omega series of polyunsaturated fatty acids and it is a potent antioxidant that has many positive effects in the body (67,68). Considering the results of this study, it can be thought that the use of lipiodol may be reasonable. Water-soluble contrast agents are associated with decreased complications and better radiographic quality compared to the lipo-soluble contrast media (69,70,71). For this reason, hydro-soluble contrast media are widely used during HSG. The result of all these studies is that there is still no definitive optimally safe method for HSG. For now, it seems more logical to use hysterosalpingo-foam sonography (HyFoSy) to evaluate tubal patency (19).

Findings of Dreyer et al. (72) suggested that in case a HyFoSy procedure is performed as the first-line tubal patency test during the fertility work-up, an HSG can be avoided in the vast majority of cases (95% confidence intervals). Perhaps HyFoSy may be considered prior to HSG as a first line assessment. In addition to the previously stated advantages of HyFoSy, the procedure appears to be less expensive than HSG. In general, HyFoSy is a less painful and less time-consuming tubal patency test compared to HSG. It also appears to be an accurate and safe test that can be performed by a single operator in an outpatient clinic setting without the need for radiation exposure, making it a far safer and more patient-friendly first-line tubal patency test (72). Future research should focus on whether tubal patency testing during the fertility workup using HyFoSy leads to the same diagnostic outcomes, subsequent management decisions, and ongoing pregnancy rates as tubal testing using HSG (73,74). To date no large trials have been published comparing HSG with HyFoSy.

## Conclusion

As seen in both previous studies and in our study, women are exposed to many harmful agents during HSG. It is therefore important that more benign and patients friendly methods should be investigated and introduced in order to optimise the evaluation of infertile women. For now, first line use of HyFoSy seems advisable with subsequent HSG if necessary, not least because HyFoSy eliminates exposure to ionizing radiation.

## Figures and Tables

**Table 1:**
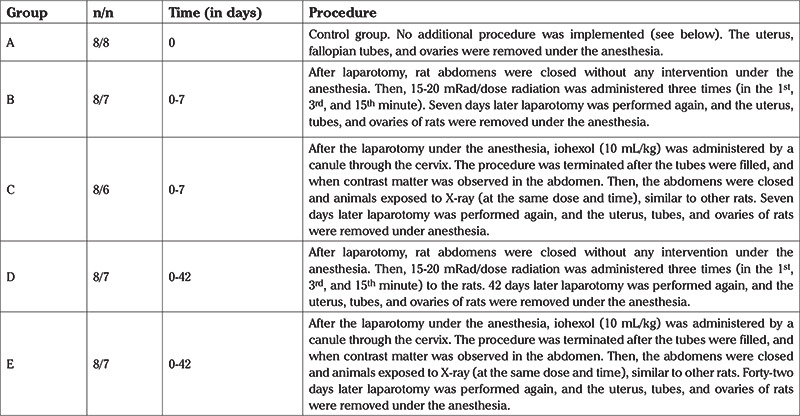
Details of experimental procedures applied to each group of animals

**Table 2:**
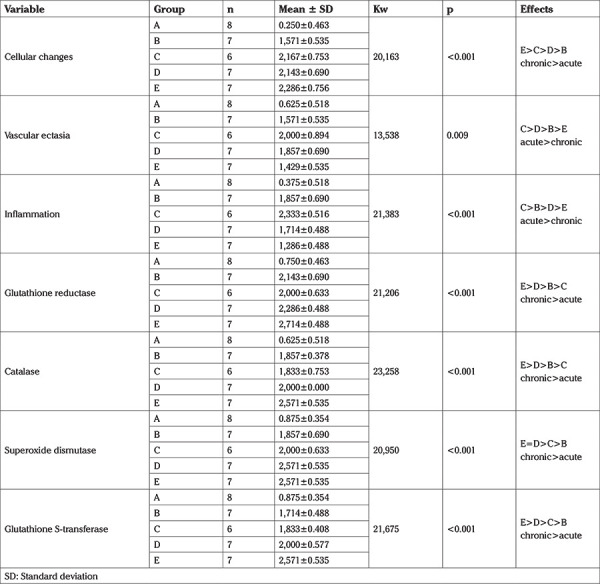
Comparison of parameters by groups
